# The complete nucleotide molecular sequence of plastid genome of *Zanthoxylum armatum* (Rutaceae)

**DOI:** 10.1080/23802359.2020.1823263

**Published:** 2020-09-22

**Authors:** Wei Sa, Jian Liang, Qian-Han Shang, Mi-Li Liu, Ma-Li Wang, Zhong-Hu Li

**Affiliations:** a State Key Laboratory of Plateau Ecology and Agriculture, College of Ecological and Environmental Engineering, Qinghai University, Xining, China; b Key Laboratory of Resources Biology and Biotechnology in Western China, Ministry of Education, College of Life Sciences, Northwest University, Xi'an, China

**Keywords:** *Zanthoxylum armatum*, plastid genome, phylogenetic analysis

## Abstract

*Zanthoxylum armatum* DC. (Rutaceae) is a shrub and/or tree species with the important medicinal and economic values. In this study, the plastid genome of *Z*. *armatum* was characterized by Illumina Hiseq 2500 sequencing platform. In total, the plastid genome is 158,557 bp in length, and comprises a large single copy region of 85,752 bp, a small single copy region of 17,605 bp, and two inverted repeat regions of 27,600 bp. The complete plastid genome contains 87 protein-coding genes, 37 tRNA genes, and 8 rRNA genes. Phylogenetic analysis suggested that *Z*. *armatum* and the congeneric *Z. simulans* clustered into an evolutionary clade with the high support.

*Zanthoxylum armatum* DC. (Rutaceae) is widely distributed in the temperate and tropics regions of east Asia. The extract of bark, fruits and seeds of *Z. armatum* is commonly used in the folklore medicine (Kalia et al. 1999). Due to multiple utilities and high cultural value, unsustainable harvest of above ground parts, and low regeneration in nature (Kala 2010), the natural population resources of *Z. armatum* has been rapidly decreased (Samant et al. 2007). It is urgent to protect and manage natural resurces of *Z. armatum.* In plant, plastid genome provided valuable phylogenetic information, owning to its conserved genome structures and comparatively high evolutionary rates (Wu and Ge 2012). In this study, we characterized the complete nucleotide sequence of plastid genome of *Z*. *armatum* (GenBank accession number: MN080708) based on the Illumina next-generation sequencing technology.

The individual of *Z*. *armatum* was collected from the Qinling Mountains (N: 33.9862; E:108.7231), Shaanxi, China. The dried plant samples and voucher specimens (No. ZALZH2018015) were deposited in the Key Laboratory of Resource Biology and Biotechnology in Western China (Shaanxi, China). The total genomic DNA was isolated from leaf materials using modified CTAB procedure (Doyle and Doyle 1987). Then, the fragmented DNAs were subjected to Illumina samples preparation, and pair-end sequenced using an Illumina Hiseq 2500 platform by Novogene Bioinformatics Technology Co., Ltd (Beijing, China). 1,065,639 reads were obtained and assembled with the program MITObim v1.8 (Hahn et al. 2013) using the congeneric *Zanthoxylum simulans* (NC_037482) as reference. The plastid genome annotation was conducted using the newly developed biosoftware CPGAVAS2 (http://47.96.249.172:16019/analyzer/home, Shi et al. 2019).

The complete plastid genome of *Z*. *armatum* was 1,58,557 bp in length and comprises a large single copy (LSC) region with 85,752 bp, a small single copy (SSC) region with 17,605 bp, and two inverted repeat regions (IRs) of 27,600 bp, which formed a typical quadripartite DNA molecular structure. The complete plastid genome contains 132 genes, including 87 protein-coding genes (PCGs), 37 transfer RNA genes (tRNA) and 8 ribosomal RNA genes (rRNA). A total of 16 genes (*atpF*, *ndhA*, *ndhB*, *petB*, *petD*, *rpl2*, *rpl16*, *rps12*, *rps16*, *rpoC1*, *trnA*-*UGC*, *trnI*-*GAU*, *trnG*-*UCC*, *trnK*-*UUU*, *trnL*-*UAA*, *trnV*-*UAC*) contained one intron, and 2 genes (*ycf3*, *clpP*) contained 2 introns. The overall GC content of *Z*. *armatum* plastid genome is 38.5%, while the corresponding values of LSC, SSC, and IR regions are 36.8, 33.6, and 42.5%, respectively.

In order to determine phylogenetic position of *Z*. *armatum*, the whole plastid genome sequences of 16 species were available downloaded from NCBI. All of the 17 plastid sequences were aligned using the software MAFFT (Katoh and Standley 2013) with the default parameter settings. We used the Modeltest v3.7 (Posada and Crandall 1998) to determine the best-fitting model. The phylogenetic analysis was conducted using the program RAxML (Stamatakis 2014) with 1000 bootstrap replicates. The results revealed that *Z*. *armatum*, *Z*. *Simulans*, *Z*. *bungeanum*, *Z*. *schinifolium* and *Z*. *piperitum* presented a group with high bootstrap support value (100%) (Figure 1).

**Figure 1.**
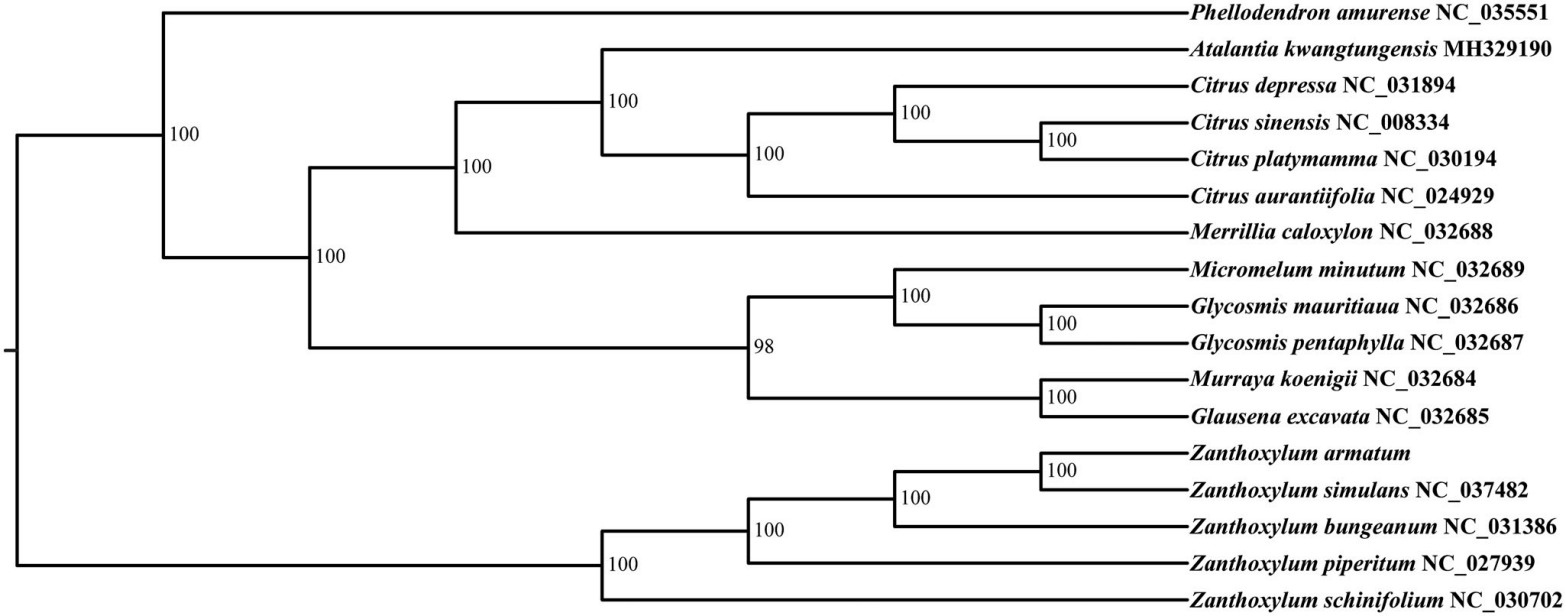
Phylogenetic tree based on seventeen nucleotide sequences of whole plastid genomes.

## Data Availability

The data that support the results of this research are openly available in GenBank at https://www.ncbi.nlm.nih.gov, reference number MN080708.
